# Trough anti-Xa activity after intermediate dose nadroparin for thrombosis prophylaxis in critically ill patients with COVID-19 and acute kidney injury

**DOI:** 10.1038/s41598-022-21560-2

**Published:** 2022-10-18

**Authors:** R. J. Eck, J. J. C. M. van de Leur, R. Wiersema, E. G. M. Cox, W. Bult, A. J. Spanjersberg, I. C. C. van der Horst, M. V. Lukens, R. O. B. Gans, K. Meijer, F. Keus

**Affiliations:** 1 Department of Internal Medicine, University Medical Center Groningen, University of Groningen, P.O. Box 30.001, 9700 RB Groningen, The Netherlands; 2 Department of Laboratory Medicine and Thrombosis Expertise Centre, Isala, Zwolle, The Netherlands; 3 Department of Critical Care, University Medical Center Groningen, University of Groningen, Groningen, The Netherlands; 4 Department of Clinical Pharmacy and Pharmacology, University Medical Center Groningen, Groningen, The Netherlands; 5 Department of Anesthesiology and Intensive Care, Isala, Zwolle, The Netherlands; 6 Department of Intensive Care, Maastricht University Medical Center+, Maastricht, The Netherlands; 7 Cardiovascular Research Institute Maastricht (CARIM), Maastricht University, Maastricht, The Netherlands; 8 Department of Laboratory Medicine, University Medical Center Groningen, University of Groningen, Groningen, The Netherlands; 9 Department of Haematology, University Medical Center Groningen, University of Groningen, Groningen, The Netherlands

**Keywords:** Adverse effects, Acute kidney injury, Epidemiology

## Abstract

Our objective was to assess the incidence of drug bioaccumulation in critically ill COVID-19 patients with AKI receiving intermediate dose nadroparin for thrombosis prophylaxis. We conducted a Prospective cohort study of critically ill COVID-19 patients. In patients on intermediate dose nadroparin (5700 IU once daily) we assessed the incidence of bioaccumulation (trough anti-Xa level > 0.2 IU/mL) stratified according to presence of AKI. We quantified this association using multilevel analyses. To assess robustness of our observations, we explored the association between AKI and anti-Xa activity in patients receiving high dose nadroparin (> 5700 IU). 108 patients received intermediate dose nadroparin, of whom 24 had AKI during 36 anti-Xa measurements. One patient with AKI (4.2% [95%CI 0.1–21%]) and 1 without (1.2% [95%CI 0.03–6.5%]) developed bioaccumulation (*p* = 0.39). Development of AKI was associated with a mean increase of 0.04 (95%CI 0.02–0.05) IU/ml anti-Xa activity. There was no statistically significant association between anti-Xa activity and AKI in 51 patients on high dose nadroparin. There were four major bleeding events, all in patients on high dose nadroparin. In conclusion, Bioaccumulation of an intermediate dose nadroparin did not occur to a significant extent in critically ill patients with COVID-19 complicated by AKI. Dose adjustment in AKI may be unnecessary.

## Introduction

Many patients with a severe course of illness of Coronavirus disease 2019 (COVID-19) require prolonged hospitalization and admittance to an Intensive Care Unit (ICU)^1^. The incidence of venous thromboembolism (VTE) in these patients is high, although point estimations vary widely^2–4^. This has prompted the conduct of several randomized clinical trials (RCTs) evaluating different prophylactic LMWH doses. The REMAP-CAP, ACTIV-4, and ATTAC multiplatform collaborative RCT concluded high dose prophylaxis is futile and may in fact be harmful compared to a standard dose in critically ill patients^5^. This was confirmed in the INSPIRATION trial that found no benefit of a weight-adjusted over a standard enoxaparin dose^6^.

The ‘standard dose’ control arm in previous RCTs included a mix of conventional low and intermediate dose LMWH, leaving uncertainty with regard to the best dose^7^. Moreover, the optimal management of critically ill patients with acute kidney injury (AKI) at high risk of thrombosis remains uncertain^8^. LMWHs are mainly excreted by the kidney and AKI may predispose to bioaccumulation with an increased risk of bleeding^9^. Faced with this dilemma, clinicians often lower the LMWH dose, a practice based on low-quality evidence^8^. Both the multiplatform collaborative RCT and the INSPIRATION trial applied LMWH dose reductions or a switch to unfractionated heparin in patients with renal failure^5,6,8^.

Since RCTs on thrombosis prophylaxis in critically ill patients were not designed nor powered to detect differences in safety outcomes in patients with AKI, there may be a role for indirect evidence to support decision-making. The anticoagulant effect of LMWHs can be monitored by measuring the ability of plasma from patients treated with LMWH to inhibit exogenous factor Xa; the resultant assay is known as anti-Xa level. Trough anti-Xa levels may reflect LMWH bioaccumulation and serve as a surrogate marker for risk of bleeding, although the optimal cut-off is uncertain^10–13^. Previous studies in critically ill patients with renal failure found no arguments for bioaccumulation of dalteparin, yet data for nadroparin are very limited despite its widespread use^9,14–16^. The objective of this study was to assess the incidence of drug bioaccumulation in critically ill patients with COVID-19 and AKI who received intermediate dose nadroparin (5700 IU once daily) for thrombosis prophylaxis.

## Methods

### Study setting

In a prospective cohort study, we included all critically ill COVID-19 patients admitted to the ICU of the University Medical Center Groningen (UMCG) between 19 March and 31 December 2020 and to the ICU of Isala between 5 April and 15 June 2020. When the first signals of a high VTE incidence emerged early in the pandemic, in both study hospitals the nadroparin dose used for prophylaxis was pragmatically increased despite the absence of data from randomized trials. As a safety precaution, routine trough anti-Xa monitoring protocols were implemented. These extraordinary circumstances enabled us to collect data on different practice patterns, covariates, and their effects on trough anti-Xa levels. Inclusion criteria were documented SARS-CoV-2 infection (a positive real-time reverse transcriptase polymerase chain reaction), age over 18 years, and treatment with nadroparin. Patients who received other anticoagulants that may influence anti-Xa levels, such as unfractionated heparin, or who explicitly refused consent for research activities, were excluded. All patients received standard supportive care and treatment. This included dexamethasone after publication of the RECOVERY trial, but generally no remdesivir, other antivirals, tocilizumab, or convalescent plasma^17^. Because we only used on anti-Xa measurements that were obtained in clinical practice, the local institutional review boards that approved the study, provided a waiver for informed consent (the Central Ethics Committee for the UMCG, 2020/221; and the Central Ethics Committee for Isala, nr. 200,629). All research was performed in accordance with relevant guidelines and regulations. This study is reported according to the strengthening the reporting of observational studies in epidemiology (STROBE) guidance^18^.

### Nadroparin dose

We used an a priori defined classification that was based on summary of product characteristics and dosing regimens of previous trials to categorize the nadroparin dose (Supplementary Table 1)^19^. Patients in the UMCG received either intermediate dose nadroparin (5700 IU once daily; Supplementary Table 1) or, in case of VTE, high dose nadroparin (5700–9500 IU twice daily adjusted for body weight; Supplementary Table 1). All patients in Isala received high dose nadroparin.

### Assessment of bioaccumulation

The objective of this study was to assess the incidence of drug bioaccumulation in critically ill patients with AKI who received intermediate dose nadroparin for thrombosis prophylaxis. We performed two analyses.

In the main analysis, we assessed trough anti-Xa levels in patients receiving intermediate dose nadroparin (Supplementary Fig. 1). Following local protocol, trough anti-Xa levels were measured twice weekly on Monday and Thursday in all patients. Measurements were performed 20–25 h after the previous dose (just before the next dose). In previous studies, trough anti-Xa activity up to 0.2 IU/ml was not associated with excessive bleeding, whereas levels > 0.2 IU/ml within twelve hours after LMWH administration were associated with higher risk of wound hematoma in orthopedic patients^10,11,14^. Accordingly, we assumed that trough anti-Xa levels above 0.2 IU/ml reflect nadroparin bioaccumulation and may serve as a marker for risk of bleeding. Since bioaccumulation is unlikely to occur before multiple doses of LMWH have been administered, we defined three consecutive administrations of intermediate dose nadroparin before trough anti-Xa measurement as a ‘stable dose course’. We assumed that trough anti-Xa levels measured after a stable dose course may be used to reliably detect or exclude bioaccumulation. We recognize that five drug half-lives are required to reach a steady-state concentration, which is why anti-Xa levels were measured twice weekly to ensure both sufficient early detection of bioaccumulation but also adequate follow-up in case bioaccumulation was delayed. The local protocol recommended lowering the nadroparin dose to 2850 IU once daily in case of trough anti-Xa levels above 0.2 IU/ml.

In the secondary analysis, we assessed trough anti-Xa levels in patients receiving high dose nadroparin (Supplementary Fig. 1). Trough anti-Xa levels were measured 10–12 h after the previous dose (just before the next dose), either by discretion of the treating physician (UMCG) or based on a local protocol (Isala). We performed this analysis because it may provide circumstantial evidence: the absence of an association between trough anti-Xa levels and AKI in patients treated with high dose nadroparin would make bioaccumulation of intermediate dose nadroparin implausible from a biological perspective. The reverse scenario, in turn, could decrease confidence in the main analysis. We defined a ‘stable dose course’ as three consecutive administrations of high dose nadroparin before anti-Xa measurement. Since anti-Xa measurements may guide high dose nadroparin (ie, lead to dose reductions / adaptations), we only used the first available measurement in each patient.

Inclusion of patients in either analysis was driven by the nadroparin dose. This meant that patients were sometimes eligible for inclusion in both analyses (Supplementary Fig. 1). For example, if a patient on intermediate dose nadroparin (eligible for inclusion in the main analysis) developed VTE and nadroparin was converted to a high dose, they were now excluded from the main analysis, but eligible for the secondary analysis, provided trough anti-Xa levels were available in both treatment periods.

### Data collection

Patient characteristics such as demographical data, medication use, comorbidities, and severity of illness scores were registered upon ICU admission. We collected daily data on nadroparin dose and timing, and registered all available anti-Xa levels. In the UMCG, anti-Xa activity measurements were performed using the Hemosil Liquid anti-Xa assay (this test does not contain exogenous antithrombin) according to the protocols from the manufacturer on a ACLTOP 500 from Werfen (Barcelona, Spain). In Isala anti-Xa activity was measured with Innovance® Heparin assay (Siemens Healthineers, Marburg, Germany) on the Symex® CS-2500 (Siemens Healthineers, the Hague, the Netherlands). In a recent study, the assay results of these manufacturers were comparable^20^. Comorbidity data were defined following the Dutch National Intensive Care Evaluation registry; specifically, chronic kidney disease (CKD) was defined by serum creatinine above 177 μmol/L before ICU admission, and a documented history of kidney disease^21^. AKI was defined according to the Kidney Disease Improving Global Outcomes (KDIGO) criteria using serum creatinine and renal replacement therapy (RRT), but not urinary output^22,23^. Baseline creatinine was not available for most patients. It was imputed assuming an estimated glomerular filtration rate (eGFR) of 75 mL/min/1.73m^2^, using the Modification of Diet in Renal Disease (MDRD) formula, except for patients who had known CKD in whom baseline creatinine was used. For each patient, the AKI stage was calculated on a daily basis to assess whether patients had AKI at the time of anti-Xa measurement. VTE was defined as any objectively proven deep vein thrombosis (DVT) or pulmonary embolism (PE) occurring during initial hospital admission. No screening protocol was used. Patients who received anticoagulant treatment based on a strong clinical suspicion of PE but in whom no objective diagnosis could be made (for example, due to inability to undergo CT angiography because of severe hemodynamic or respiratory instability) were also registered to have a VTE. Major bleeding was defined according to the International Society on Thrombosis and Haemostasis criteria^24^. Follow-up was until hospital discharge, transfer to another hospital, or mortality.

### Statistical analysis

Data are presented as means with standard deviations (SD) or medians with interquartile ranges [IQR] depending on distributions. Categorical data are presented as proportions with 95% confidence intervals (CI) calculated using the Clopper-Pearson interval. Groups were compared using the Fisher’s exact test, t-test or Mann–Whitney U-test depending on distributions. We performed no formal sample size calculation.

For the main analysis (to assess the incidence of bioaccumulation in patients receiving intermediate dose nadroparin) we calculated the proportion (95% CI) of patients with at least one trough anti-Xa level > 0.2 IU/mL in two groups: those who had anti-Xa measurements while having any episode of AKI, and those without AKI. Analyses were not further stratified according to AKI stage due to the low sample size. To quantify the association between trough anti-Xa levels and renal function, we used a linear multilevel model with trough anti-Xa levels modelled as a function of time and AKI, adjusted for body mass index (BMI). Analyses were limited to patients with a stable dose course of intermediate dose nadroparin. AKI was entered into the model as a time-dependent binary variable to allow for non-linear change over time. We assessed interaction terms between the covariates (AKI and BMI) and time. Clustering in the data was accounted for by a random intercept and random slope at patient level. We selected the best fitting model based on likelihood ratio tests. Model assumptions were checked and judged to have been sufficiently met. Since trough anti-Xa values below the limit of detection are reported as ‘ < 0.04 IU/ml’ we performed the analyses twice: with imputed values of 0.0 IU/ml (main model) and 0.02 IU/ml (sensitivity analysis 1). To explore the effect of any recent nadroparin dose change we did a second sensitivity analysis including all patients regardless of recent dose changes (sensitivity analysis 2).

For the secondary analysis, we compared trough anti-Xa levels in patients with AKI to anti-Xa levels in patients without AKI using a Mann–Whitney U test. A two-sided *P*-value < 0.05 indicated statistical significance. All analyses were performed using R software version 4.0.4.

### Ethics approval and consent to participate

The local institutional review boards provided a waiver for informed consent (the Central Ethics Committee for the UMCG, 2020/221; and the Central Ethics Committee for Isala, nr. 200,629).

## Results

In total, 234 patients were assessed for eligibility (Supplementary Fig. 1). Eighty-six patients were excluded either because they received continuous heparin or other anticoagulant interventions (n = 9), or no trough anti-Xa levels were available (n = 77) (Supplementary Fig. 1). Baseline characteristics were comparable between in- and excluded patients but clinical outcomes did show differences in length of stay.

(Supplementary Table 2). Characteristics for the 108 patients who were included in the main analyses are presented in Table 1 and for 51 patients who were included in the secondary analyses in Supplementary Table 3. Eleven patients were included in both analyses (Supplementary Fig. 1). Overall, most patients were on invasive mechanical ventilation within 24 h after ICU admission. Seven patients had CKD; none were on chronic dialysis. During ICU and subsequent hospital stay, 32 patients developed VTE (21.9%; 95% CI 15.5–29.5%). Four patients who received high dose nadroparin developed major bleeding (2.7%; 95% CI 0.7–6.8%). No patient developed major bleeding while on intermediate dose nadroparin. The amount of missing data was low.

**Table 1:** Main analysis: characteristics and clinical outcomes.

	No AKI (n = 84)	AKI (n = 24)
**Baseline characteristics**		
Age in years, mean (SD)	63.6 (7.9)	63.1 (9.6)
Sex, female, n (%)	27 (32.1%)	6 (25.0%)
BMI, mean (SD)	29.9 (4.5)	29.5 (4.5)
APACHE IV score, mean (SD)	56.0 (16.0)	62.7 (14.5)
* Missing, n (%)*	*5 (5.9%)*	*0 (0%)*
Invasive mechanical ventilation within 24 h, n (%)	74 (88.1%)	23 (95.8%)
Peak anti-Xa activity, mean (SD)*	0.21 (0.10)	0.14 (0.10)
* Missing, n (%)*	*71 (84.5%)*	*21 (87.5%)*
**Comorbidities**		
Chronic kidney disease, n (%)	4 (4.8%)	3 (12.5%)
Dialysis, n (%)	0 (0.0%)	0 (0.0%)
Diabetes mellitus, n (%)	23 (27.4%)	6 (25.0%)
Hypertension, n (%)	31 (36.9%)	14 (58.3%)
Chronic heart failure, n (%)	0 (0%)	0 (0%)
Any previous venous thrombotic event, n (%)	0 (0%)	1 (4.2%)
Any previous major bleeding even, n (%)	0 (0%)	0 (0%)
**Clinical outcomes**		
ICU length of stay in days, median [IQR]	10.7 [7.8 -15.2]	15.7 [9.3–22.1]
Hospital length of stay in days, median [IQR]	17.7 [12.8–27.9]	21.9 [16.6–28.5]
VTE, n (%)	14 (16.9%)	9 (37.5%)
* Missing, n (%)*	*1 (1.2%)*	*0 (0%)*
Major bleeding, n (%)^#^	1 (1.2%)	2 (8.3%)
* Missing, n (%)*	*1 (1.2%)*	*0 (0%)*
In-hospital mortality, n (%)	16 (19.3%)	14 (58.3%)
* Missing, n (%)*	*1 (1.2%)*	*0 (0%)*

*APACHE* Acute physiology and chronic health evaluation, *BMI* body mass index, *ICU* Intensive care unit, *IQR* interquartile range, *n* number, *SD* standard deviation, *VTE* venous thromboembolism.

*Calculation of mean peak anti-Xa activity was based on the highest peak anti-Xa measurement in 16 patients on intermediate dose nadroparin.

^#^All bleeding events occurred after intermediate dose nadroparin had been converted to high dose nadroparin, for example for atrial fibrillation or VTE.

Missing values are in [italics].

### Main analysis

**Figure 1:**
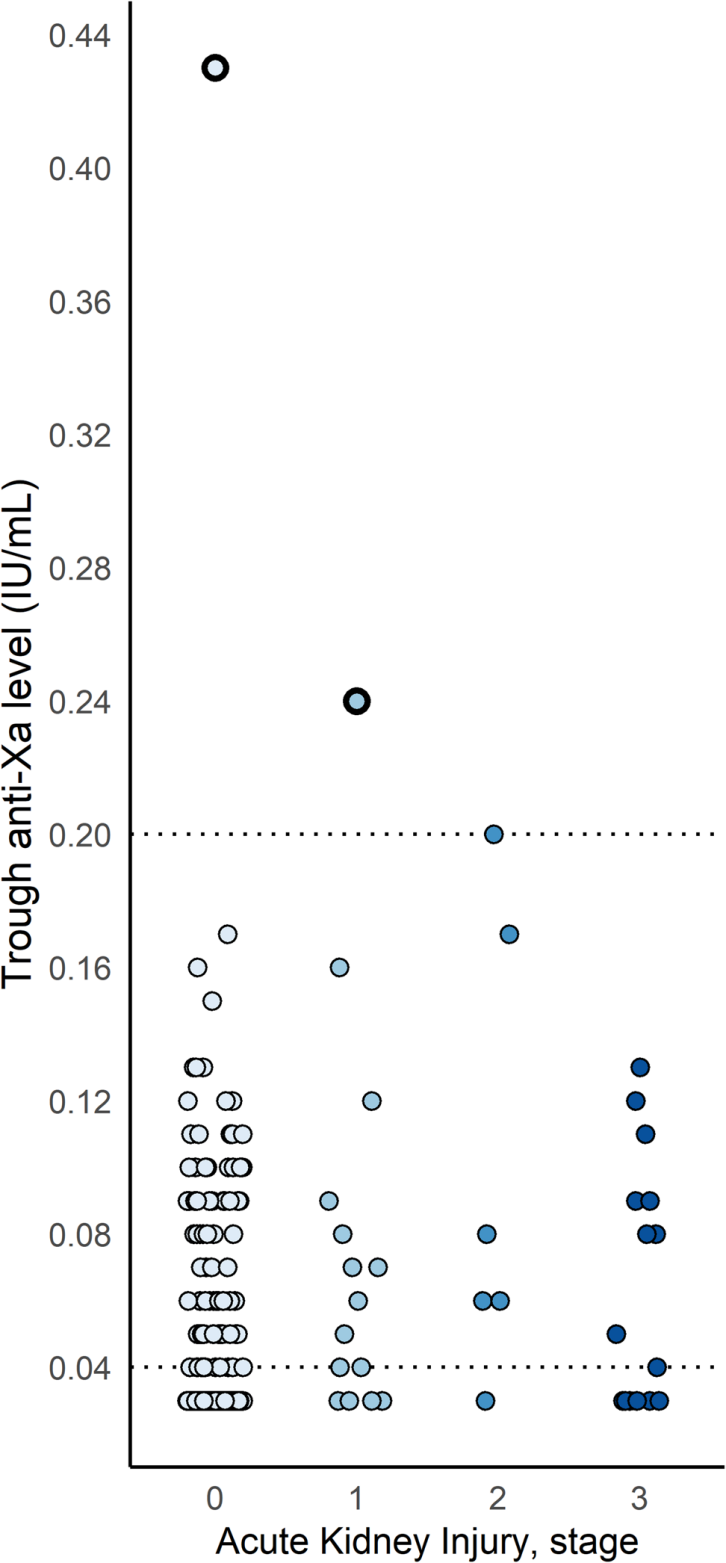
Trough anti-Xa levels according to the presence of AKI (includes clustered data). The horizontal dotted lines indicate the respective cut-off points for the limit of detection (< 0.04) and our definition of bioaccumulation (> 0.20 IU/ml). The plot shows 234 trough anti-Xa levels of 108 patients who received intermediate dose nadroparin. The plot is intended for a general overview of the data and includes clustered data, so no summary statistics are provided.

**Figure 2:**
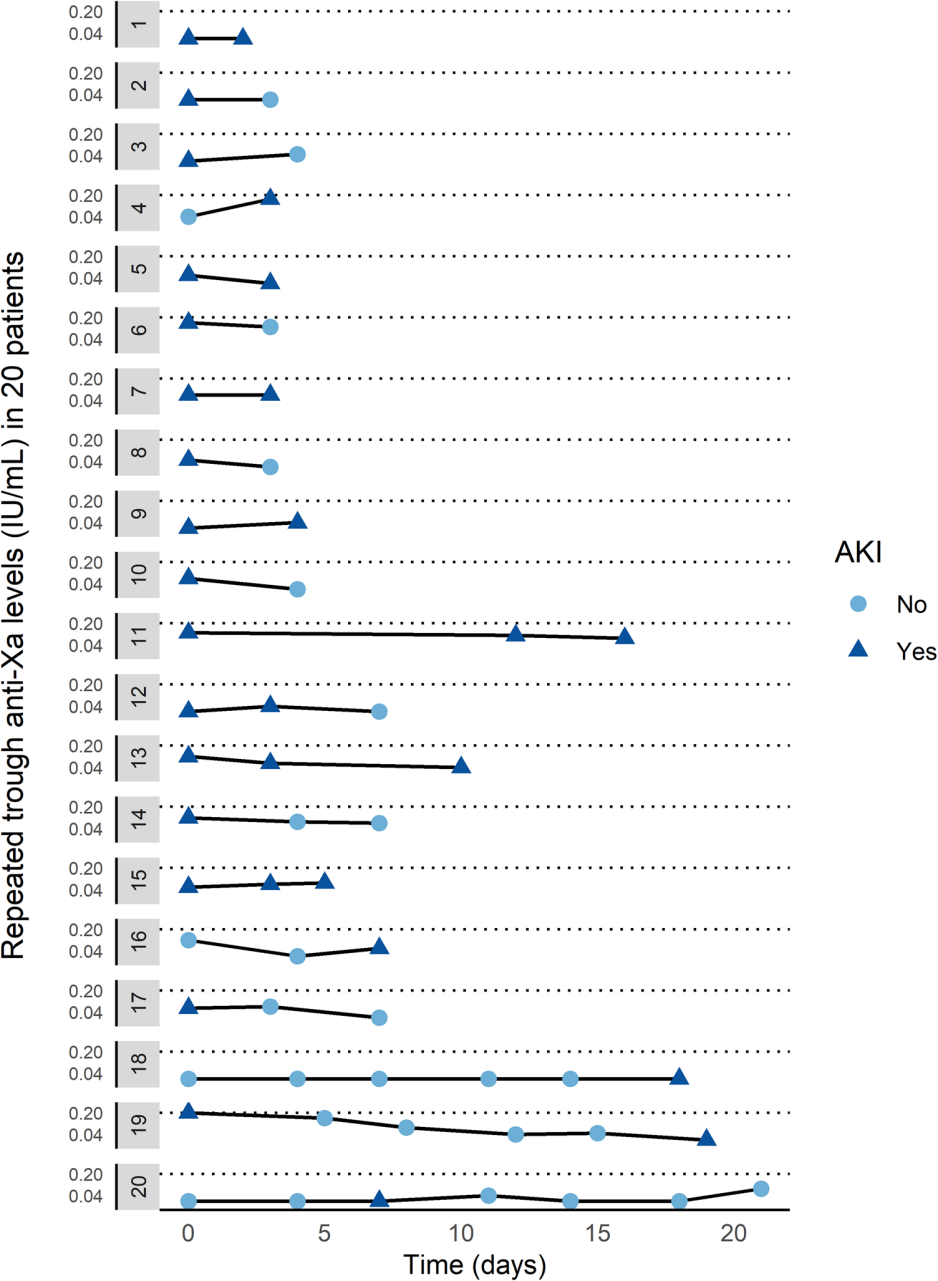
Trough anti-Xa levels in 20 patients with repeated measurements and AKI. Each horizontal line represents a patient receiving intermediate dose nadroparin while having repeated anti-Xa measurements, either during an episode of AKI (triangle) or no AKI (circle). The number of measurements for each patient varied from 2 to 7. The horizontal dotted lines indicate the cut-off point for our definition of bioaccumulation (> 0.20 IU/ml). AKI: acute kidney injury.

A total of 234 trough anti-Xa measurements were obtained in 108 patients receiving intermediate dose nadroparin, resulting in a median of 2 (IQR 1–3) measures per patient. Twenty-four (22.2%) patients had AKI during one or more of the measurements, resulting in 36 (15.4%) trough anti-Xa measurements in the presence of AKI (Fig. 1). The development of trough anti-Xa levels over time is shown for 20 patients with AKI and repeated measurements in Fig. 2 and for all patients with repeated measurements in Supplementary Fig. 2. One included patient was treated with renal replacement therapy. In both groups 1 patient developed bioaccumulation: incidence of 4.2% (95% CI 0.1–21%) for patients with AKI versus 1.2% (95% CI 0.03–6.5%) for those without AKI (*p* = 0.39; Fig. 1). The anti-Xa level normalized after dose reduction in the patient with AKI (who also had pre-existing CKD) and without dose reduction in the other. Overall, there was a slight decrease of anti-Xa levels over time with 0.002 (0.001–0.003) IU/ml per day, while the development of AKI was associated with a small mean increase of 0.04 (0.02–0.05) IU/ml (Table 2). Including BMI did not substantially improve the model (Table 2). The sensitivity analysis estimates were similar, albeit slightly lower (Table 2). Overall, 210 trough anti-Xa levels (89.7%) had been measured after a stable course of intermediate dose nadroparin, while 13 (5.6%) had been measured after patients had received one or more higher doses, eight (3.4%) after one or more lower doses, and data for three (1.3%) patients were missing.

**Table 2:** Results from multilevel analyses.

Model	Model fit (AIC)	Parameter	Estimate (95% CI)	*P*-value
Main model	− 723.8	Time in days	− 0.002 (− 0.003 to − 0.001)	< 0.01
		AKI (yes/no)^a^	0.04 (0.02 to 0.05)	< 0.01
Main model + BMI + BMI*time	− 723.8	Time in days	0.002 (− 0.005 to 0.008)	0.65
		AKI (yes/no)^a^	0.04 (0.02 to 0.05)	< 0.01
		BMI	0.0007 (− 0.03 to 0.004)	0.71
		BMI*Time in days	− 0.0001 (− 0.0004 to 0.00009)	0.25
Sensitivity analysis 1	− 801.6	Time in days	− 0.002 (− 0.003 to − 0.001)	< 0.01
		AKI (yes/no)^a^	0.03 (0.02 to 0.05)	< 0.01
Sensitivity analysis 2	− 785.8	Time in days	− 0.002 (− 0.003 to − 0.001)	< 0.01
		AKI (yes/no)^a^	0.03 (0.02 to 0.05)	< 0.01

*AIC* Akaike Information Criterion, *AKI* Acute Kidney Injury, *BMI* body mass index.

^a^AKI was entered in the models as a time-dependent dichotomous variable.

### Secondary analysis

There were 51 trough anti-Xa measurements in 51 patients treated with high dose nadroparin. Of these, 12 measurements (23.5%) were performed in the presence of AKI (Supplementary Fig. 3). Median trough anti-Xa values were 0.27 (0.17–0.42) IU/ml in patients with AKI and 0.28 (0.20–0.41) IU/ml in patients without AKI (*p* = 0.67). Thirty-six (70.6%) anti-Xa levels had been measured after a stable dose course, while 2 (3.9%) had been measured after patients had received one or more higher doses, eight (19.6%) after one or more lower doses, and data for three (5.9%) patients were missing.

## Discussion

In critically ill patients with COVID-19 receiving intermediate dose nadroparin for thrombosis prophylaxis, the incidence of bioaccumulation was low regardless of the presence of AKI. Longitudinal data analysis suggested a small increase in trough anti-Xa activity when patients developed AKI, but this increase was of low clinical relevance. These observations were further supported by the fact that median trough anti-Xa levels in patients treated with high dose nadroparin who had AKI were not increased compared to patients without AKI. The overall incidence of VTE in the entire cohort was high (21.9%; 95% CI 15.5–29.5%) and none of the patients on intermediate dose nadroparin developed a major bleeding event. Together, these data suggest that adjustment of intermediate dose nadroparin to prevent bioaccumulation in critically patients with COVID-19 and AKI may not be necessary, and even inappropriate considering the high VTE proportion.

This study represents one of the first reports on the incidence of nadroparin bioaccumulation in critically ill patients with AKI. Previous studies in patients with COVID-19 and AKI were (understandably) focused on efficacy instead of safety^25,26^. Aside from the COVID-19 literature, current evidence on risk of bioaccumulation in patients with AKI differs per LMWH type^9^. Each LMWH has its own pharmacokinetic properties, limiting exchangeability of bioaccumulation data^27^. In two cohorts of patients with severe renal failure there was no evidence for bioaccumulation of dalteparin^14,16^. In contrast, studies in volunteers and hospitalized elderly medical patients have suggested bioaccumulation of enoxaparin occurs when the creatinine clearance is below 30 ml/min^12,13,28^. In line with our findings, an observational study in medical patients with moderate renal insufficiency found no evidence for accumulation of intermediate dose nadroparin^15^. Data on high dose nadroparin are limited, with only one previous study describing an inverse correlation between eGFR and high dose nadroparin^9^. Given our data and the results from literature, bioaccumulation of either low or intermediate dose nadroparin in critically ill patients with AKI is probably rare, while uncertainty remains on high (ie therapeutic) doses.

In judging whether our findings are generalizable to patients without COVID-19, we should consider one important confounder: the relatively high mean BMI of the patients in our cohort. Previous studies have shown an inverse association between BMI and peak anti-Xa activity, presumably due to a reduced subcutaneous absorption^29^. This could suggest that patients with a lower BMI would have had higher trough anti-Xa levels. However, measured peak anti-Xa levels were in line with a previous report on COVID-19 patients and with a large body of existing literature in general critically ill patients, both of which have shown that LMWH prophylaxis is generally associated with low peak anti-Xa levels^30–34^. Given this comparability, we have no strong arguments to assume that trough anti-Xa levels were systematically lower in our cohort, and results may be generalizable to critically ill patients suffering from other conditions.

The main strengths of this study include the measurement of trough anti-Xa levels according to a predefined local protocol irrespective of the presence of AKI or other patient characteristics, mitigating the potential for selection bias. We registered data on nadroparin dose and the presence of AKI daily to avoid misclassification errors. Finally, most trough anti-Xa levels were measured after a minimum three-day course of intermediate dose nadroparin, which should allow for reliable detection or exclusion of bioaccumulation. There are several limitations to our study. First, the estimated incidence of bioaccumulation was imprecise due to the low number of patients with AKI, and the confidence interval was compatible with a meaningful increased risk of bioaccumulation. However, this limitation is partly mitigated because we applied a conservative threshold for defining bioaccumulation (studies have used higher cut-offs of > 0.4 IU/mL), so even higher trough anti-Xa levels may still be associated with an acceptable bleeding risk^14^. Second, we did not adjust the longitudinal analyses for vasopressor use, which is associated with decreased anti-Xa levels and may act as a potential confounder^32^. Third, the low sample size precluded more elaborate statistical analyses of the association between anti-Xa levels and VTE and bleeding outcomes. Fourth, in several eligible patients no trough anti-Xa levels were measured. This was most likely because the measurement protocol was not sufficiently implemented in some of our hospital’s improvised ICU wards at the peak of the pandemic. We excluded these cases from our study assuming missingness was completely at random, but of course this assumption is hard to validate. Fifth, baseline creatinine was estimated for most patients, decreasing precision of the AKI estimates. This limitation, however, affects most studies on AKI in the ICU as this is the most commonly employed method^23^. Finally, we used trough anti-Xa activity as a putative surrogate for risk of bleeding, but the strength of the underlying association is uncertain.

In conclusion, bioaccumulation of an intermediate dose nadroparin did not occur to a significant extent in critically ill patients with COVID-19 complicated by AKI. Accordingly, dose adjustment in AKI may not be necessary. These results ought to be generalizable to general critically ill patients with the caveat that patients with low to normal BMI were underrepresented in our cohort.

## Supplementary Information

Supplementary Information.

## Data Availability

The datasets used in the current study are available from the corresponding author on reasonable request.
