# Supplementary material for: Trough anti-Xa activity after intermediate dose nadroparin for thrombosis prophylaxis in critically ill patients with COVID-19 and acute kidney injury
Source: Sci Rep. 2022 Oct 18;12:17408. doi: 10.1038/s41598-022-21560-2 (PMC9579123; doi:10.1038/s41598-022-21560-2)
Supplement: Supplementary file 1 — Supplementary Information. [file 41598_2022_21560_MOESM1_ESM.docx]

Supplementary appendix

**Trough anti-Xa activity after intermediate dose nadroparin for thrombosis prophylaxis in critically ill patients with COVID-19 and acute kidney injury**

R.J. Eck^1^, J.J.C.M. van de Leur^2^, R. Wiersema^3^, E.G.M. Cox^3^, W. Bult^3,4^, A.J. Spanjersberg^5^, I.C.C. van der Horst^6,7^, M.V. Lukens^8^, R.O.B. Gans^1^, K. Meijer^9^, F. Keus^3^

**Author affiliations and departments**

1 Department of Internal Medicine, University Medical Center Groningen, University of Groningen, The Netherlands

2 Department of Laboratory Medicine and Thrombosis Expertise Centre, Isala, Zwolle, the Netherlands

3 Department of Critical Care, University Medical Center Groningen, University of Groningen, The Netherlands

4 Department of Clinical Pharmacy and Pharmacology, University Medical Center Groningen, Groningen, The Netherlands

5 Department of Anesthesiology and Intensive Care, Isala, Zwolle, the Netherlands

6 Department of Intensive Care, Maastricht University Medical Center+, The Netherlands

7 Cardiovascular Research Institute Maastricht (CARIM), Maastricht University, Maastricht, the Netherlands

8 Department of Laboratory Medicine, University Medical Center Groningen, University of Groningen, The Netherlands

9 Department of Haematology, University Medical Center Groningen, University of Groningen, The Netherlands

| Supplementary table 1. Classification of low-molecular-weight heparin dose | | | |
| --- | --- | --- | --- |
| Product | **Dose** | | |
|  | **Low** | **Intermediate** | **High** |
| Nadroparin | 2850 IU qd | 5700 IU qd | 171 IU /kg qd or 86 IU /kg bid |
| Dalteparin | 2500 IU qd | 5000 IU qd | 200 IU /kg qd or 100 IU /kg bid |
| Enoxaparin | 20 or 30 mg qd | 40 mg qd or 30 mg bid | 1,5 mg /kg qd or 1 mg /kg bid |
| Tinzaparin | 3500 IU qd | 4500 IU qd | 175 IU /kg qd |
| IU: International Units; mg: milligrams; qd: once daily; bid: twice daily. Nadroparin is the drug of interest for the current study. This table is not exhaustive (several other types of LMWHs exist). Dalteparin, enoxaparin, and tinzaparin are displayed for illustrative purposes, so readers may compare nadroparin dose to other LMWH types they are more familiar with. The dosing categories were based on multiple international summary of product characteristics (1–8). | | | |

| Supplementary Table 2. Comparison of in- and excluded patients | | | |
| --- | --- | --- | --- |
| Baseline characteristics | **Included patients**  **n = 148** | **Excluded patients**  **n = 86** | **P-value** |
| Age in years, mean (SD) | 63.5 (8.9) | 62.5 (10.9) | 0.46 |
| Sex, female, n (%) | 41 (27.7%) | 23 (26.7%) | 0.99 |
| BMI, mean (SD) | 29.7 (4.3) | 29.2 (4.9%) | 0.49 |
| APACHE IV score, mean (SD)  *Missing, n (%)* | 58.3 (15.5)  *5 (3.4%)* | 61.0 (19.0)  *3 (3.5%)* | 0.26 |
| Invasive mechanical ventilation within 24h, n (%) | 137 (92.6%) | 76 (88.4%) | 0.40 |
|  |  |  |  |
| Comorbidities | |  |  |
| Chronic kidney disease, n (%) | 7 (4.7%) | 4 (4.7%) | 1.00 |
| Dialysis, n (%) | 0 (0.0%) | 0 (0.0%) | NA |
| Diabetes mellitus, n (%) | 36 (24.3%) | 25 (29.1%) | 0.52 |
| Hypertension, n (%) | 58 (39.2%) | 44 (51.2%) | 0.10 |
| Chronic heart failure, n (%) | 0 (0%) | 2 (2.3%) | 0.13 |
| Any previous venous thrombotic event, n (%) | 6 (4.1%) | 3 (3.5%) | 1.00 |
| Any previous major bleeding event, n (%) | 0 (0%) | 0 (0%) | NA |
|  |  |  |  |
| Clinical outcomes | |  |  |
| ICU length of stay, days, median [IQR] | 14.0 [8.4 – 20.7] | 9.4 [5.4 - 18.4] | **0.001** |
| Hospital length of stay, days, median [IQR] | 20.8 [14.6 - 30.9] | 14.8 [8.8 - 21.9] | **<0.001** |
| VTE, n (%)  *Missing, n (%)* | 32 (21.9%)  *2 (1.4%)* | 15 (17.6%)  *1 (1.2%)* | 0.54 |
| Major bleeding, n (%)  *Missing, n (%)* | 4 (2.7%)  *2 (1.4%)* | 6 (7.1%)  *1 (1.2%)* | 0.18 |
| In-hospital mortality, n (%)  *Missing, n (%)* | 39 (27.3%)  *5 (3.4%)* | 26 (30.6%)  *1 (1.2%)* | 0.70 |
| APACHE: Acute Physiology And Chronic Health Evaluation; BMI: body mass index; ICU: Intensive Care Unit; IQR: interquartile range; n = number; NA: not applicable; SD: standard deviation; VTE: venous thromboembolism | | | |

| Supplementary Table 3. Secondary analysis: characteristics and clinical outcomes | | |
| --- | --- | --- |
|  | **No AKI (n = 39)** | **AKI (n = 12)** |
| Baseline characteristics |  |  |
| Age in years, mean (SD) | 63.8 (9.9) | 61.3 (8.5) |
| Sex, female, n (%) | 9 (23.1%) | 1 (8.3%) |
| BMI, mean (SD) | 29.0 (4.2) | 29.5 (2.7) |
| APACHE IV score, mean (SD)  *Missing, n (%)* | 58.7 (13.8) | 63.0 (16.5) |
| Invasive mechanical ventilation within 24h, n (%) | 37 (94.9%) | 12 (100%) |
|  |  |  |
| Comorbidities |  |  |
| Chronic kidney disease, n (%) | 1 (2.6%) | 1 (8.3%) |
| Dialysis, n (%) | 0 (0%) | 0 (0%) |
| Diabetes mellitus, n (%) | 9 (23.1%) | 3 (25.0%) |
| Hypertension, n (%) | 14 (35.9%) | 6 (50.0%) |
| Chronic heart failure, n (%) | 0 (0%) | 0 (0%) |
| Any previous venous thrombotic event, n (%) | 3 (7.7%) | 2 (16.7%) |
| Any previous major bleeding even, n (%) | 0 (0%) | 0 (0%) |
|  |  |  |
| Clinical outcomes |  |  |
| ICU length of stay in days, median [IQR] | 17.0 [11.9 - 25.5] | 21.8 [15.4 - 41.0] |
| Hospital length of stay in days, median [IQR] | 26.0 [17.7 - 33.4] | 33.8 [26.5 - 51.7] |
| VTE, n (%)  *Missing, n (%)* | 10 (26.3%)  *1 (2.6%)* | 6 (50.0%)  *0 (0%)* |
| Major bleeding, n (%)  *Missing, n (%)* | 2 (5.3%)  *1 (2.6%)* | *0 (0%)*  *0 (0%)* |
| In-hospital mortality, n (%)  *Missing, n (%)* | 9 (25.0%)  3 (7.7%) | 4 (36.4%)  *0 (0%)* |
|  |  |  |
| APACHE: Acute Physiology And Chronic Health Evaluation; BMI: body mass index; ICU: Intensive Care Unit; IQR: interquartile range; n = number; SD: standard deviation; VTE: venous thromboembolism. | | |

**Supplementary figure 1. Flow chart of patients included in main and secondary analysis**


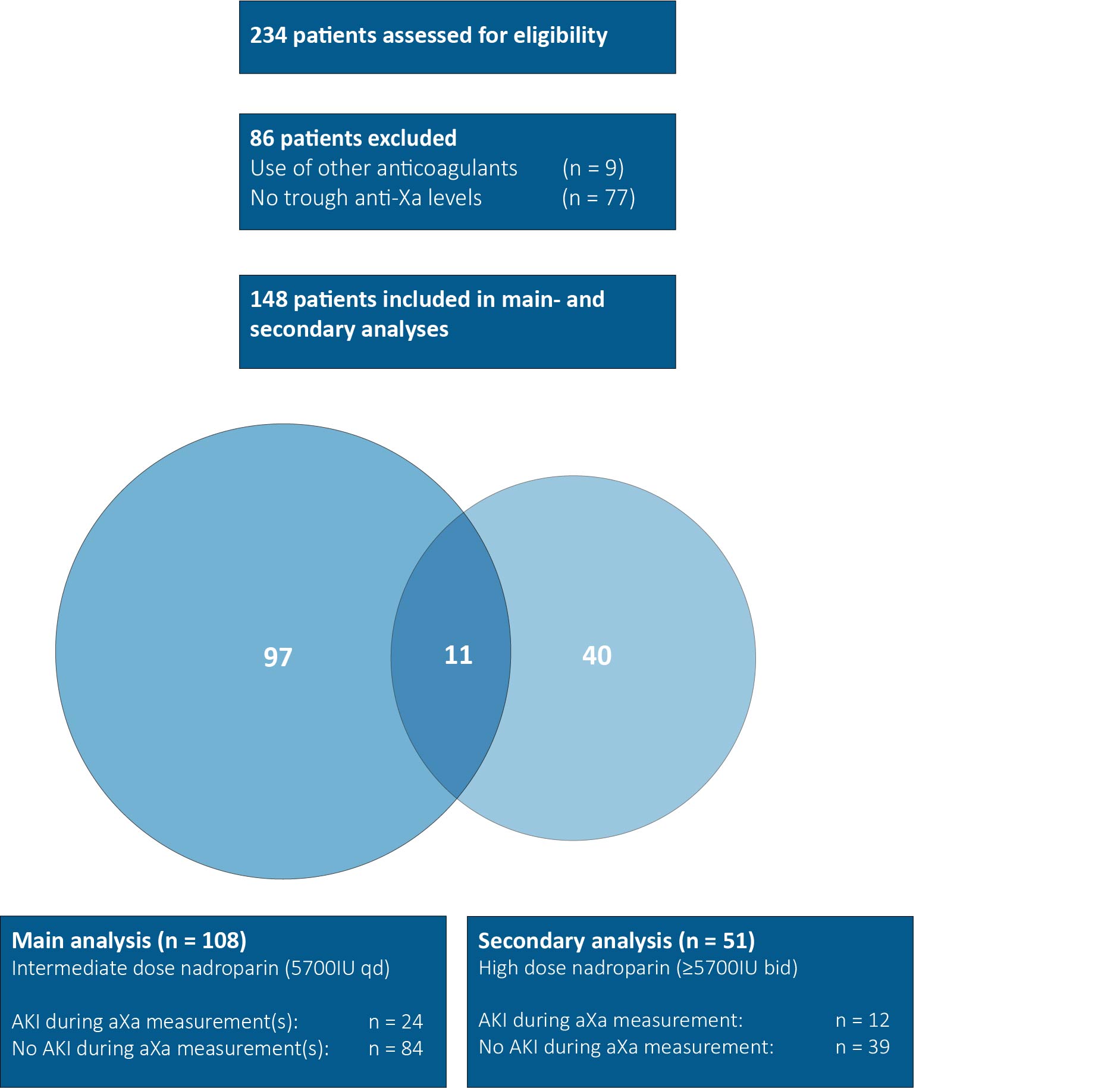
AKI: Acute Kidney Injury; aXa: Anti-Xa activity; bid: twice daily; qd: once daily.

**
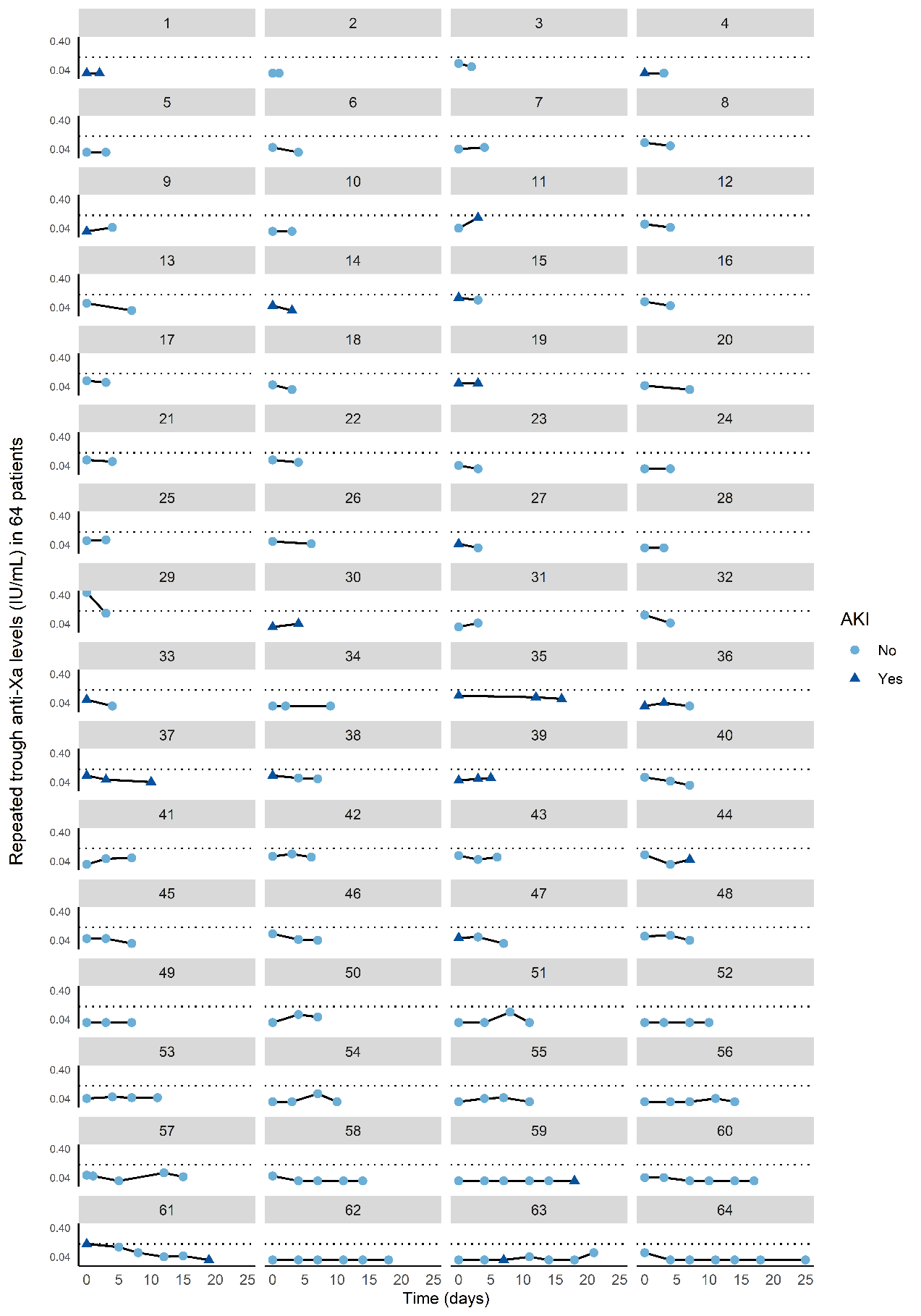
Supplementary figure 2. Trough anti-Xa levels in all patients with repeated measurements**

Each horizontal line represents a patient receiving intermediate dose nadroparin while having repeated anti-Xa measurements, either during an episode of AKI (triangle) or no AKI (circle). The number of measurements for each patient varied from 2 to 7. The horizontal dotted lines indicate the cut-off point for our definition of bioaccumulation (>0.20 IU/ml).

**Supplementary figure 3. Trough anti-Xa measurements in patients receiving high dose nadroparin, according to presence of AKI.**

**
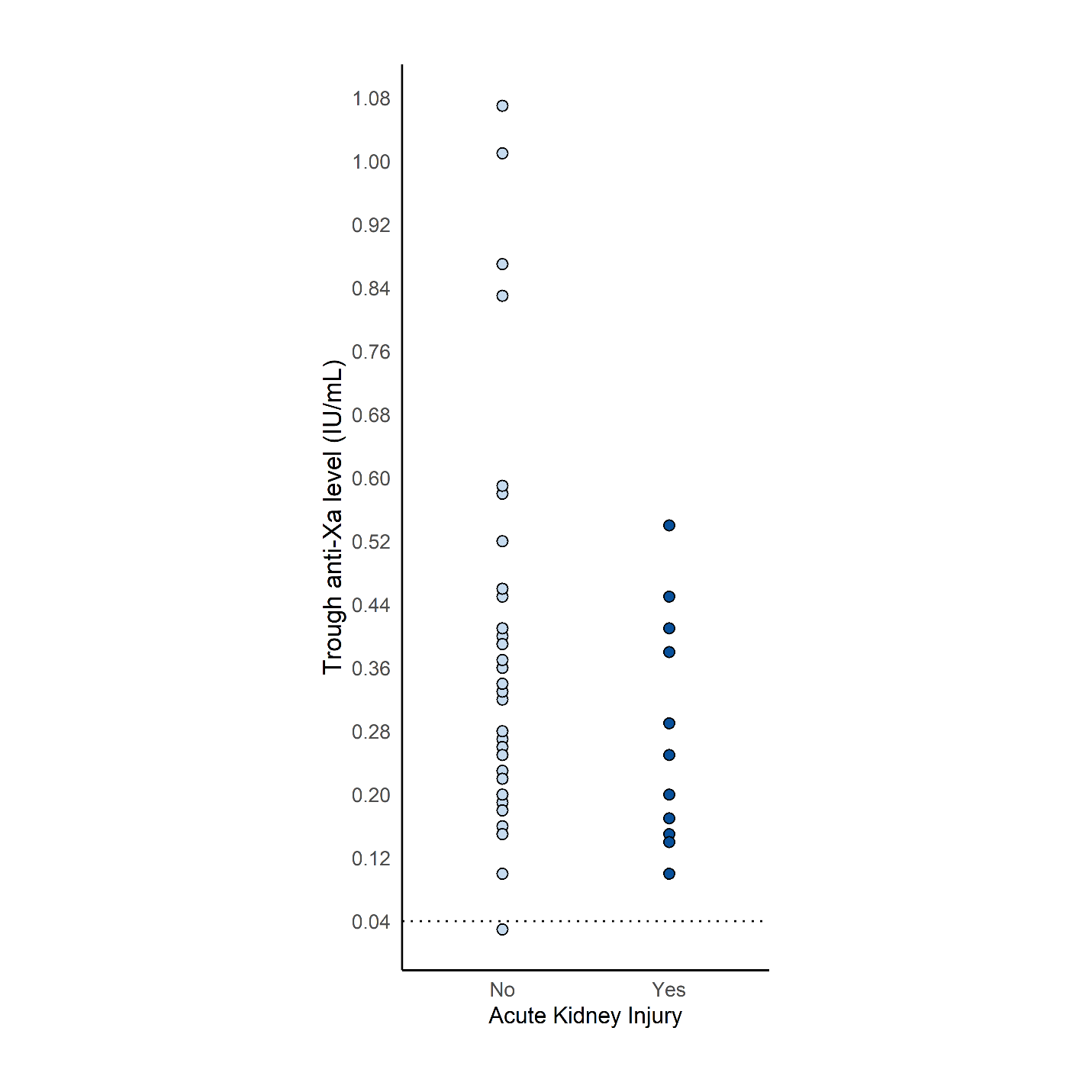
**

The horizontal dotted line indicates the cut-off point for limit of detection (<0.04 IU/ml).

**Supplementary references**

1. Food and Drug Administration (United States). Drug approval reports [Internet]. [cited 2019 Feb 7]. Available from: www.accessdata.fda.gov/scripts/cder/daf/

2. Federal Institute for Drugs and Medical Devices (Germany). Summary of Product Characteristics [Internet]. [cited 2016 Jan 31]. Available from: www.bfarm.de/DE/Home/home_node.html

3. The Israeli Drug Registry (Israel). Summary of product characteristics. [Internet]. [cited 2019 Feb 7]. Available from: https://data.health.gov.il/drugs/index.html#/byDrug

4. Italian Medicines Agency (Italy). Summary of Product Characteristics [Internet]. [cited 2019 Feb 7]. Available from: www.agenziafarmaco.gov.it/en

5. National Agency for the Safety of Medicine and Health Products (France). Summary of Product Characteristics [Internet]. [cited 2019 Feb 7]. Available from: www.ansm.sante.fr/Produits-de-sante/Medicaments

6. Medicines & Healthcare products Regulatory Agency (United Kingdom). Summary of Product Characteristics [Internet]. [cited 2017 Jul 31]. Available from: http://www.mhra.gov.uk/spc-pil/

7. Medicines Evaluation Board (The Netherlands). Summary of Product Characteristics [Internet]. [cited 2019 Feb 7]. Available from: www.geneesmiddeleninformatiebank.nl/nl/

8. Health Products Regulatory Authority (Ireland). Summary of Product Characteristics [Internet]. [cited 2019 Feb 7]. Available from: www.hpra.ie/homepage/medicines/medicines-information/find-a-medicine
